# Simulated Microgravity Potentiates Hematopoietic Differentiation of Human Pluripotent Stem Cells and Supports Formation of 3D Hematopoietic Cluster

**DOI:** 10.3389/fcell.2021.797060

**Published:** 2022-01-10

**Authors:** Chiyuan Ma, Yue Xiong, Pei Han, Xueying Zhang, Yujing Cao, Baobei Wang, Huashan Zhao, Enkui Duan, Jian V. Zhang, Xiaohua Lei

**Affiliations:** ^1^ Center for Energy Metabolism and Reproduction, Shenzhen Institutes of Advanced Technology, Chinese Academy of Sciences, Shenzhen, China; ^2^ Technology and Engineering Center for Space Utilization, Chinese Academy of Sciences, Beijing, China; ^3^ State Key Laboratory of Stem Cell and Reproductive Biology, Institute of Zoology, Chinese Academy of Sciences, Beijing, China; ^4^ Shenzhen Institutes of Advanced Technology, Chinese Academy of Sciences, Shenzhen, China

**Keywords:** human embryonic stem cells, hematopoietic differentiation, microgravity, 3D clusters, transcriptome analysis

## Abstract

Microgravity has been shown to induces many changes in proliferation, differentiation and growth behavior of stem cells. Little is known about the effect of microgravity on hematopoietic differentiation of pluripotent stem cells (PSCs). In this study, we used the random position machine (RPM) to investigate whether simulated microgravity (SMG) allows the induction of hematopoietic stem/progenitor cell (HSPC) derived from human embryonic stem cells (hESCs) *in vitro*. The results showed that SMG facilitates hESCs differentiate to HSPC with more efficient induction of CD34^+^CD31^+^ hemogenic endothelium progenitors (HEPs) on day 4 and CD34^+^CD43^+^ HSPC on day 7, and these cells shows an increased generation of functional hematopoietic cells in colony-forming unit assay when compared with normal gravity (NG) conditions. Additionally, we found that SMG significantly increased the total number of cells on day 4 and day 7 which formed more 3D cell clusters. Transcriptome analysis of cells identified thousands of differentially expressed genes (DEGs) between NG and SMG. DEGs down-regulated were enriched in the axonogenesis, positive regulation of cell adhesion, cell adhesion molecule and axon guidance, while SMG resulted in the up-regulation of genes were functionally associated with DNA replication, cell cycle, PI3K-Akt signaling pathway and tumorigenesis. Interestingly, some key gene terms were enriched in SMG, like hypoxia and ECM receptor interaction. Moreover, HSPC obtained from SMG culture conditions had a robust ability of proliferation *in vitro*. The proliferated cells also had the ability to form erythroid, granulocyte and monocyte/macrophage colonies, and can be induced to generate macrophages and megakaryocytes. In summary, our data has shown a potent impact of microgravity on hematopoietic differentiation of hPSCs for the first time and reveals an underlying mechanism for the effect of SMG on hematopoiesis development.

## Introduction

Exposure to microgravity induces a variety of changes in physiology and some specialized tissue, including musculoskeletal system, nervous system, cardiovascular system, and immune system (Garrett-Bakelman et al., 2019; Bradbury et al., 2020). Previous studies have suggested that microgravity also provides a unique environment for stem cell growth, differentiation and metabolism change (Lei et al., 2014; Lei et al., 2018; Costantini et al., 2019; Grimm et al., 2020). Some studies demonstrated that the negative effect of microgravity on the differentiation of stem cells, such as microgravity attenuates osteoblasts differentiation (Chen et al., 2016; Zhang et al., 2018) and myogenic differentiation (Furukawa et al., 2018). Other studies have also shown that microgravity can promote the hepatic differentiation in stem cells (Wang et al., 2012; Lu et al., 2019). However, the effect of microgravity on hematopoietic differentiation of human embryonic stem cells (hESCs) has not been yet examined.

hESCs have the capacity of self-renewal and multipotent differentiation, which can offer an invaluable tool for tracking early human hematopoietic development and produce hematopoietic stem/progenitor cell (HSPC) and functional blood cells for therapies of various hematologic disorders. It has been reported that three critical stages of development are required in the specification of hematovascular precursors from hESCs, including mesoderm specifications, hemogenic endothelium progenitors (HEPs) induction and HSPC emergence (Slukvin, 2013). Several signaling pathways were identified to play vital roles in hematopoietic differentiation of hESCs. The activation of canonical Wnt signaling with GSK-3 inhibitor CHIR99021 in hESCs can enhance definitive hematopoiesis and decrease the number of primitive HSPC (Sturgeon et al., 2014). Inhibition of Nodal/Activin pathway with SB-431542 in hematopoietic differentiation of hESCs can also induce the development of definitive HSPC and attenuate the primitive hematopoiesis process (Kennedy et al., 2012). Additionally, inhibition of transforming growth factor-*β* (TGF-*β*) have been reported to play a vital role in priming hemogenic potential in epithelial cells signaling at the HEPs stage are increased a capacity to produce HSPC through the downregulation of mesenchymal genes (Wang Y. et al., 2020). Although biochemical stimuli factors are known for enhancing cell fate transitions in hematopoiesis process, there is growing appreciation on the role of biomechanical stimuli factors (North et al., 2009) or through a combination of biochemical and biomechanical factors for hematopoietic differentiation (Yang et al., 2016).

The aim of the present study was to determine whether simulated microgravity effect on the hematopoietic differentiation of human embryonic stem cells (hESCs) and what is the molecular characteristics of simulated microgravity on the cells during hematopoietic differentiation. The results showed that hESCs cultured in SMG gave rise to significantly higher populations of CD31^+^CD34^+^ HEPs on day 4 and CD34^+^CD43^+^ HSPC on day 7 than those cultured in NG, and these cells showed an increased generation of functional hematopoietic cells by colony-forming unit assay, which is suggesting microgravity increases early hematopoietic differentiation derived from hESCs. Additionally, SMG significantly increased the total number of cells on day 4 and day 7 which formed more 3D cell clusters. Together, our data shows a potent impact of microgravity on hematopoietic differentiation of hPSCs for the first time and reveals an underlying mechanism for the effect of SMG on hematopoiesis development.

## Materials and Methods

### hESCs Culture and HSPC Differentiation

**FIGURE 1:**
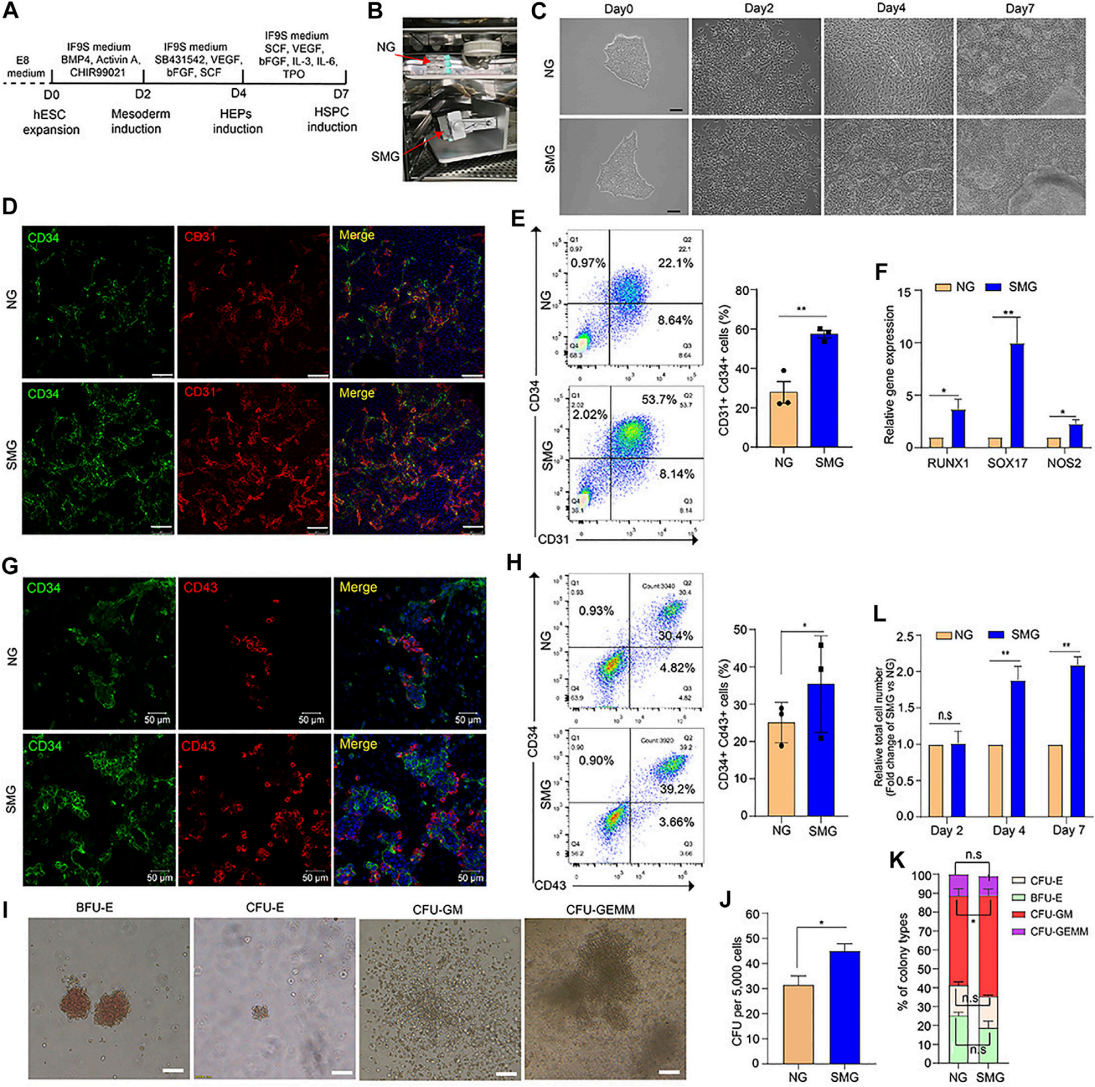
Simulated microgravity promotes hematopoietic differentiation of hESCs. **(A)** Schematic illustration of hematopoietic differentiation protocol from hESCs. **(B)** Custom-made random position machine (RPM) for the hematopoietic differentiation of hESCs to simulated microgravity conditions (SMG), the normal gravity (NG) cultivation is a control group. **(C)** Morphological change of cells during differentiation from hESCs to HSPC under NG and SMG condition on day 0, day 2, day 4 and day 7. Scale bars represent 100 μm. **(D)** Representative immunostaining images of day 4 cells for CD34 (green) and CD31 (red) under NG and SMG condition. Scale bar represents 100 μm. **(E)** Representative flow cytometry results of surface marker CD34 and CD31 in NG and SMG group at day 4, the bar graph showing the number of CD34^+^CD31^+^ hemogenic endothelium progenitors derived from NG and SMG group at day 4. **(F)** qRT-PCR analysis of RUNX1, SOX17 and NOS2 expression in the day 4 cells differentiated under NG or SMG condition. **(G)** Representative immunostaining images of day 7 cells for CD34 (green) and CD43 (red) under NG and SMG condition. Scale bar represents 50 μm. **(H)** Representative flow cytometry results of surface marker CD34 and CD43 in NG and SMG group at day 7, the bar graph showing the number of CD34^+^CD43^+^ hematopoietic stem/progenitor cells derived from NG and SMG group at day 7. **(I)** Representative colony morphologies from hESC-derived hematopoietic cells on day 7. **(J)** The total number of colonies derived from NG group and SMG group. **(L)** The percentage of BFU-E, CFU-E, CFU-GM, and CFU-GEMM in total colonies. **(K)** The relative fold change of total cell number generated in the day 4 and day 7 (SMG vs NG). For statistical significance, *p < 0.05, **p < 0.01 and n. s., not significant (*p* > 0.05), all data are presented as mean ± SEM.

For hESCs culture, the H1 hESCs were routinely maintained on Matrigel coated plates (Corning) in TeSR-E8 medium (STEMCELL Technologies) according to product specification. For hESCs hematopoietic differentiation, we used the protocol established by Cao et al. with modification (Cao et al., 2019). Briefly, undifferentiated hESCs cultured in TeSR-E8 medium were digested into small clumps of cells by ReLeSRTM (STEMCELL Technologies) and plated onto growth factor reduced Matrigel-coated (Corning) T12.5 flask at a density of 100 clumps/cm^2^ in TeSR-E8 medium. After 24 h, hESC clones were prepared for hematopoietic differentiation by three step inductions (Figure 1A). From day 0 to day 2, cells were cultured in IF9S medium supplemented with 50 ng/ml BMP4 (R&D Systems), 10 ng/ml ACTIVIN A (Miltenyi Biotec), and 1 mM CHIR99021 (STEMCELL Technologies) for 48 h. On day 2, cells were refreshed with IF9S medium supplemented with 50 ng/ml VEGF165 (R&D Systems), 20 ng/ml bFGF (PeproTech), 10 μM SB431542 (R&D Systems), and 20 ng/ml SCF (R&D Systems) for 48 h culturing. On day 4 to day 7, cells were cultured in IF9S medium supplemented with 50 ng/ml VEGF165 (R&D Systems), 50 ng/ml SCF (R&D Systems), 20 ng/ml bFGF (PeproTech), 20 ng/ml IL-6 (R&D Systems), 10 ng/ml IL-3 (R&D Systems), and 20 ng/ml TPO (Sino Biological). For cell expansion, on day 8 and day 9, floating cells were collected, analyzed or transferred into low-attachment plates and cultured in StemSpan™ medium with 50 ng/ml SCF, 50 ng/ml Flt-3 ligand, 20 ng/ml IL-6 and 20 ng/ml TPO. For adherent cells and hematopoietic clusters were further cultured in IF9S medium supplemented with 50 ng/ml VEGF165 (R&D Systems), 50 ng/ml SCF (R&D Systems), 20 ng/ml IL-6 (R&D Systems), 10 ng/ml IL-3 (R&D Systems), and 20 ng/ml TPO (Sino Biological). The entire differentiation process was incubated at 37°C in 5% CO_2_ with 100% humidity under NG or SMG conditions.

### Immunofluorescence Assays

Cells were fixed with a 4% paraformaldehyde solution at room temperature for 20 min, then permeabilized in 0.2% Triton X-100 (Sigma), blocked in 5% donkey serum (abcam) and then incubated with primary antibodies against human Brachyury/T (1:100; Santa Cruz), CD31 (1:50, BD), CD34 (1:50, BD), CD43 (1:50, BD), CD45 (1:50, BD) at 4°C overnight. These were then incubated with Alexa Fluor^®^ 488 or 568-conjugated antibodies (ThermoFisher Scientific). Nuclei were then stained with Hoechst33342 (0.1–1 μg/ml, Sigma). Confocal fluorescence microscope was used for image acquisition.

### Flow Cytometry Analysis

The differentiated cells in T12.5 flask were dissociated into single cells using 0.05% trypsin supplemented with 0.1% EDTA. The cells were washed, resuspended in a FACS washing buffer (PBS with 5% BSA and 2.5 mM EDTA) and then stained with the desired antibodies. The antibodies used in our study were: PE Mouse Anti-Human CD31 (1:25, BD), FITC Mouse Anti-Human CD34 (1:25, BD), APC Mouse Anti-Human CD34 (1:25, BD), APC Mouse Anti-Human CD43 (1:25, BD), APC Mouse Anti-Human CD45 (1:25, BD), FITC-conjugated mouse IgG2a (1:25, BD), APC-conjugated mouse IgG1 (1:25, BD) and PE-conjugated mouse IgG1κ (1:25, BD) were used as isotype-matched negative controls. Flow cytometry was performed on Calibur flow cytometer (BD) or CytoFLEX V2-B4-R2 Flow Cytometer (BECKMAN COULTER), and the data was analyzed using FlowJo software, version 10.0.7.

### RNA Isolation, Real-Time PCR

Total RNA was extracted from cells using RNeasy Mini Kit (Qiagen) according to the product manual. RNA of each sample was reverse-transcribed with Superscript III (TransGen). Real-time PCR was performed using a GoTaq qRT-PCR master mix (Promega, Madison, WI, United States) on a Roche LightCycler 480 system (Indianapolis, IN, United States). The sample input was normalized against the critical threshold (Ct) value of GAPDH. Primers sequences are listed in Supplementary Table S1.

### RNA-Sequencing and Analysis

The SMG and NG differentiated cells were collected for high-throughput RNA sequencing (RNA-seq). The RNA-seq was performed by Annoroad Gene Technology Co. Ltd (Beijing). Differentially expressed genes (DEGs) were identified using DESeq2 v1.6.3. DESeq2, to calculate the expression level of each gene per sample by using the linear regression, and the *p*-value was calculated with the Wald test. The *p*-value was then corrected by the BH method. Genes with *q* ≤ 0.05 and |log2_ratio|≥1 were identified as DEGs. The GO (Gene Ontology, http://geneontology.org/) and KEGG (http://www.kegg.jp/) enrichment of DEGs was implemented by the use of hypergeometric test, in which *p*-value was calculated and the resulting *p*-value was subjected to correction to calculate the False Discovery Rate (FDR) or adjusted *q*-value. Gene set enrichment analysis (GSEA) soft (http://software.broadinstitute.org/gsea/index.jsp) was used conduct functional gene sets enrichment analysis.

### Hematopoietic Colony-Formation Assay

The hematopoietic CFU assay was performed according the manufacturer’s instructions of MethoCult SF H4636 (STAMCELL Technologies). Differentiated HEPs or HSPCs of the indicated numbers in 0.1 ml IF9S were mixed with 2 ml MethoCultsf H4636. The mixture was then transferred to one well of ultra-low attachment 12-well plates (Corning). The cells were incubated at 37°C in 5% CO_2_ with 100% humidity for 14 days, and the colonies were counted. Each type of colony was classified according to technical manual of human colony-forming unit (CFU) assays using MethoCult™.

### Macrophage Differentiation

Differentiated HSPC were plated on 12-well tissue culture plates at a density of 20,000 cells/cm^2^ in IF9S medium supplemented with 25 ng/ml IL-3 and 50 ng/mL M-CSF for 4 days. After 4 days, medium was replaced with 50 ng/mL M-CSF for another 4 days of culture. For staining, the differentiated macrophages were centrifuged onto slides using cytospin before staining with Giemsa staining solution. The morphology was observed by microscopy.

### Megakaryocyte Differentiation

Differentiated HSPC were plated on 24-well tissue culture plates at a density of 10,000 cells/well in StemSpan™ medium supplemented with 50 ng/ml TPO, 20 ng/ml SCF, 10 ng/ml IL-3 and 20 ng/ml IL-6 for 4 days. After 4 days, medium was replaced with 50 ng/ml TPO, 5 ng/ml SCF and 20 ng/ml IL-11 for another 6 days of culture.

### Statistical Analysis

The statistical analyses were performed using GraphPad Prism 5 (GraphPad Software, La Jolla, CA). Unless stated otherwise, the results are shown as the mean ± s. e.m. One-way ANOVA or Student’s t test was used to determine the level of significance. *p* < 0.05 is considered statistically significant, and *p* < 0.01 is considered statistically very significant.

## Results

### Simulated Microgravity Culture Potentiates Hematopoiesis *in vitro*

To identify whether microgravity could affect the hematopoietic differentiation potential of hESCs, a chemically defined 3-step hematopoietic differentiation protocol (Cao et al., 2019) was used in our study (Figure 1A). The SM-31 random position machine (RPM) (National Center of Space Science, Chinese Academy of Sciences) was applied to simulated microgravity conditions, (Figure 1B and Supplementary Video S1), and cells cultured in the static condition were used in parallel as a normal gravity (NG) control. As shown in Figure 1C, the cell morphology changed rapidly in both the NG and SMG groups during 8 days of differentiation. Compared with SMG and NG, the mesenchymal like cells on day 2 were similar morphologically (Figure 1C) and the differentiation efficiency of Brachyury^+^ cells in SMG or NG group was not significant (Supplementary Figure S1B,C). However, cells exposed in SMG exhibits a more apparently endothelium-like characteristics and prone to form 3D endothelial clusters on day 4 (Figure1C). On day 7, more hematopoietic-like cells with round shape and 3D hematopoietic clusters were clearly observed in SMG group (Figure 1C and Supplementary Videos S2,3).

To determine whether SMG promoted HEP development, we detected CD34^+^CD31^+^ cells on day 4 by immunofluorescence staining and flow cytometry. Compared with NG group, SMG significantly augmented the frequency and number of CD31^+^ CD34^+^ cells (Figures 1D,E). qRT-PCR analysis showed that the expression of RUNX1, SOX17 and NOS2 was significantly higher in SMG cells than in NG cells (Figure 1F), which have been reported to play a vital role in priming hemogenic potential in endothelial cells (Nakajima-Takagi et al., 2013; Ran et al., 2013).

To further determine the differentiation potential of HSPC, we detected the expression of CD34 and CD43. The results showed that the SMG generated higher population (Figure 1G) and percentage (Figure 1H) of CD34^+^CD43^+^ cells compared with those in NG group on day 7. Additionally, we also confirmed significantly increased total numbers of hematopoietic colonies in CFU assay from SMG differentiated HSPC (Figures 1I,J), including the proportion of multipotent granulocyte/macrophage (GM) colonies (Figure 1K). Interestingly, we found that SMG significantly increased the total number of cells on day 4 and day 7 (Figure 1L) compared with NG cells. Thus, our results suggest that SMG may promotes early hematopoietic differentiation from hESCs.

### RNA-Seq Analysis of Cells During Hematopoietic Differentiation Under NG and SMG Conditions

**FIGURE 2:**
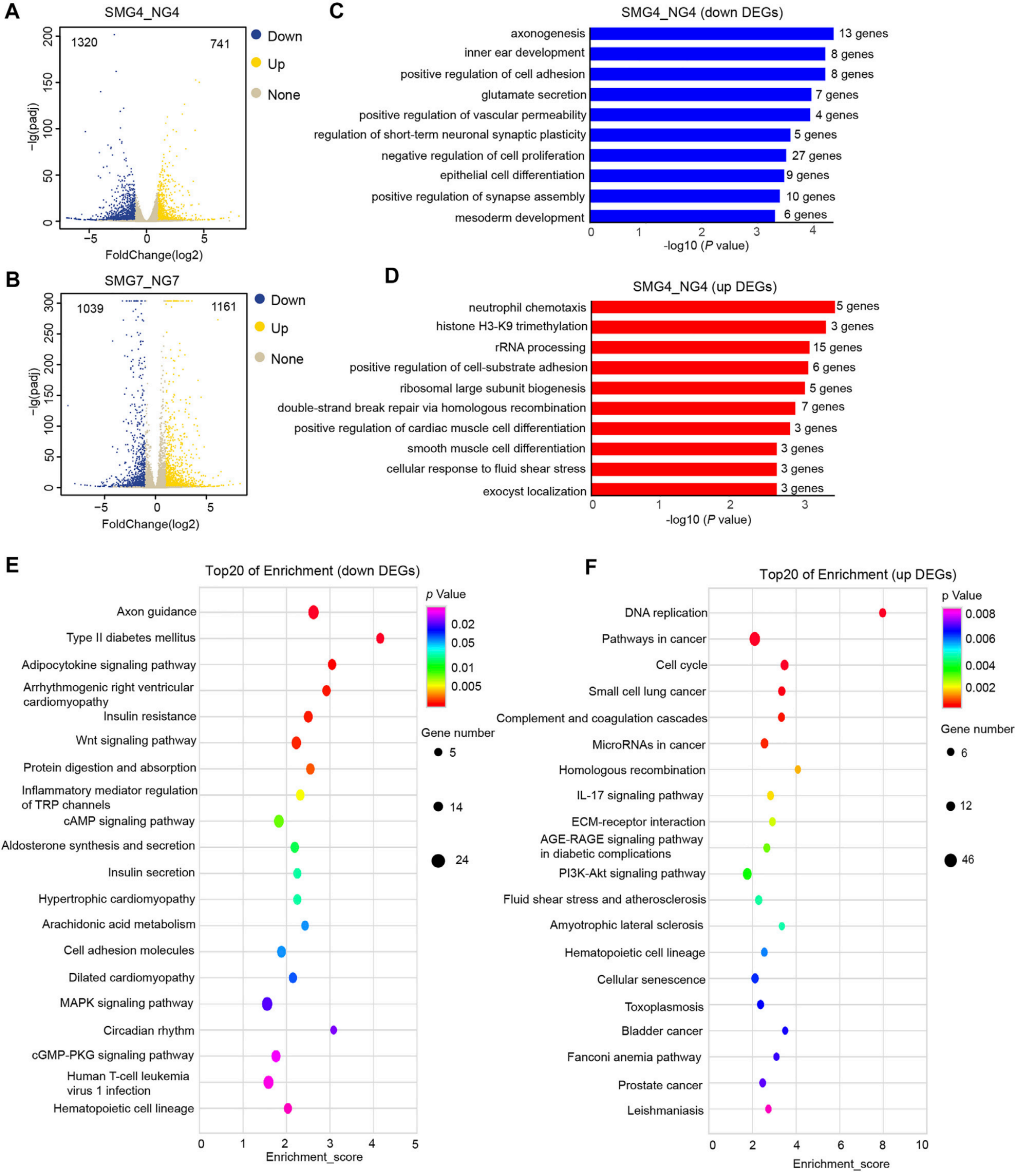
Genome -wide RNA-seq assays to detect differentially expressed genes (DEGs), GO and KEGG pathways analysis of DEGs in SMG4 vs NG4 cells. **(A)** Volcano plot displaying the DEGs in SMG or NG cells on day 4. The blue, yellow and grey dots represent down-regulated, up-regulated and not significant gene, respectively. **(B)** Volcano plot displaying the DEGs in SMG or NG cells on day 7. **(C)** Bar chart shows the top10 down-regulated GO terms of BP in SMG4 vs NG4. **(D)** Bar chart shows the top10 up-regulated GO terms of BP in SMG4 vs. NG4. **(E)** Dot plot shows the down-regulated KEGG pathways enriched in SMG4 vs NG4. The size of the dot is based on gene number enriched in the pathway, and the color of the dot shows the pathway enrichment significance. **(F)** Dot plot shows the up-regulated KEGG pathways enriched in SMG4 vs NG4. NG4, cell induced for 4 d under normal gravity condition; SMG, cell induced for 4 d under simulated microgravity condition; NG7, cell induced for 7 d under normal gravity condition; SMG, cell induced for 7 d under simulated microgravity condition.

To investigate how simulated microgravity impacted the hematopoietic differentiation potential of hESCs. We performed the genome-wide RNA-seq to detect the gene expression profiles of cells on day 4 and day 7 differentiation from the NG group and SMG group. Differentially expressed genes (DEGs) were obtained by pairwise comparisons of differentiated cells on days 4 (SMG4 vs NG4) and 7 (SMG7 vs NG7), respectively. A total of 2061 DEGs were identified in SMG4 vs NG4 cells (741 DEGs up-regulated and 1,320 DEGs down-regulated) (Figure 2A). A total of 2,200 DEGs were identified in SMG7 vs NG7 cells (1,161 DEGs up-regulated and 1,030 DEGs down-regulated) (Figure 2B).

Next we performed gene ontology (GO) enrichment analysis of identified DEGs to reveal the biological processes (BP). On day 4, DEGs down-regulated were enriched in the axonogenesis, positive regulation of cell adhesion, inner ear morphogenesis, glutamate secretion and negative regulation of cell proliferation (Figure 2C), whereas up-regulated DEGs were enriched in neutrophil chemotaxis, histone H3-K9 trimethylation, rRNA processing, positive regulation of cell-subunit biogenesis and ribosomal large subunit biogenesis (Figure 2D). Additionally, in the kyoto encyclopaedia of genes and genomes enrichment (KEGG) analysis, 20 top significant pathways were plotted in the bubble diagram, which showed that these down regulated DEGs were mainly involved in axon guidance, adipocytokine signaling pathway, insulin resistance, Wnt signaling pathway and protein digestion and absorption (Figure 2E). While DEGs up-regulated were mainly in the DNA replication, pathways in cancer, cell cycle, ECM-receptor interaction, PI3K-Akt signaling pathway and hematopoietic cell lineage-related pathways (Figure 2F).

**FIGURE 3:**
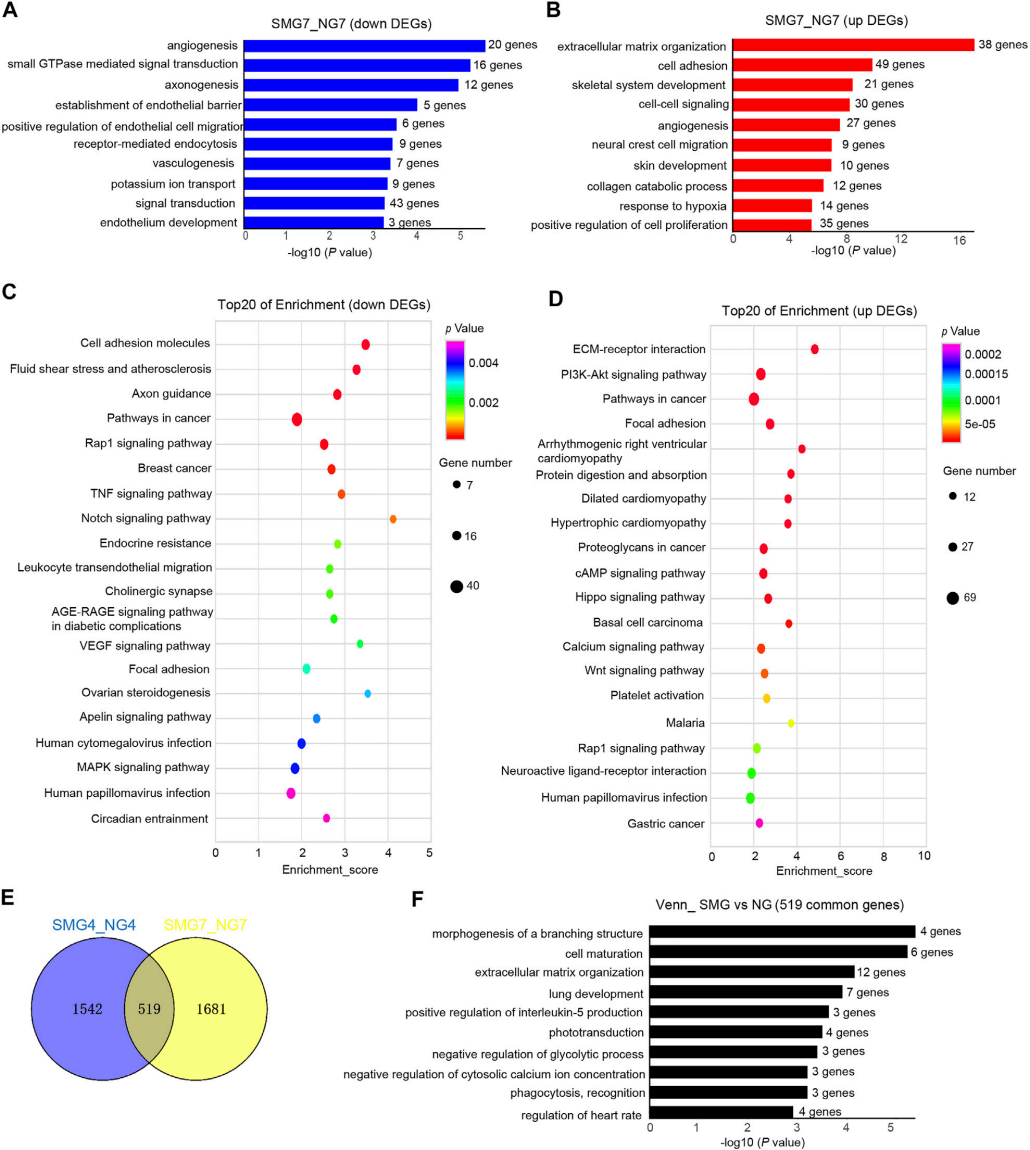
GO and KEGG pathway analysis of DEGs in SMG7 vs NG7 cells. **(A)** Bar chart shows the top10 down-regulated GO terms of BP in SMG7 vs NG7. **(B)** Bar chart shows the top10 up-regulated GO terms of BP in SMG7 vs NG7. **(C)** Dot plot shows the down-regulated KEGG pathways enriched in SMG7 vs. NG7. The size of the dot is based on gene number enriched in the pathway, and the color of the dot shows the pathway enrichment significance. **(D)** Dot plot shows the up-regulated KEGG pathways enriched in SMG7 vs. NG7. **(E)** Venn diagram of overlapping DEGs comparing SMG4 vs. NG4 and SMG7 vs. NG7. **(F)** GO analysis of overlapping DEGs between SMG4 vs NG4 and SMG7 vs NG7.

To study the hematopoiesis, DEGs on day 7 (SMG7 vs NG7) were also chosen to perform GO and KEGG analysis. In down regulated DEGs, 10 of the top enriched GO terms were related to angiogenesis, axonogenesis, establishment of endothelial barrier, positive regulation of endothelial cell migration, vasculogenesis, endothelium development pathways (Figure 3A). The up regulated DEGs enriched pathways were extracellular matrix organization, cell adhesion, angiogenesis, collagen catabolic process, response to hypoxia and positive regulation of cell proliferation (Figure 3B). Analysis of KEGG showed that down regulated DEGs were enriched in cell adhesion molecule, axon guidance, Rap1 signaling pathway, TNF signaling pathway and Notch signaling pathway (Figure 3C). The enrichment of up regulated DEGs were mainly in ECM-receptor interaction, PI3K-Akt signaling pathway, pathways in cancer, focal adhesion, protein digestion and absorption, cAMP signaling pathway, Hippo signaling pathway and Wnt signaling pathway (Figure 3D). We also found 519 overlapping DESs between SMG4 and NG on day 4 and day 7 (Figure 3E). The overlapping DEGs enriched pathways were morphogenesis of a branching structure, cell maturation, extracellular matrix organization, lung development, positive regulation of interleukin-5 production and phototransduction (Figure 3F).

### Key Gene Terms Enriched in SMG Condition During Hematopoietic Differentiation

**FIGURE 4:**
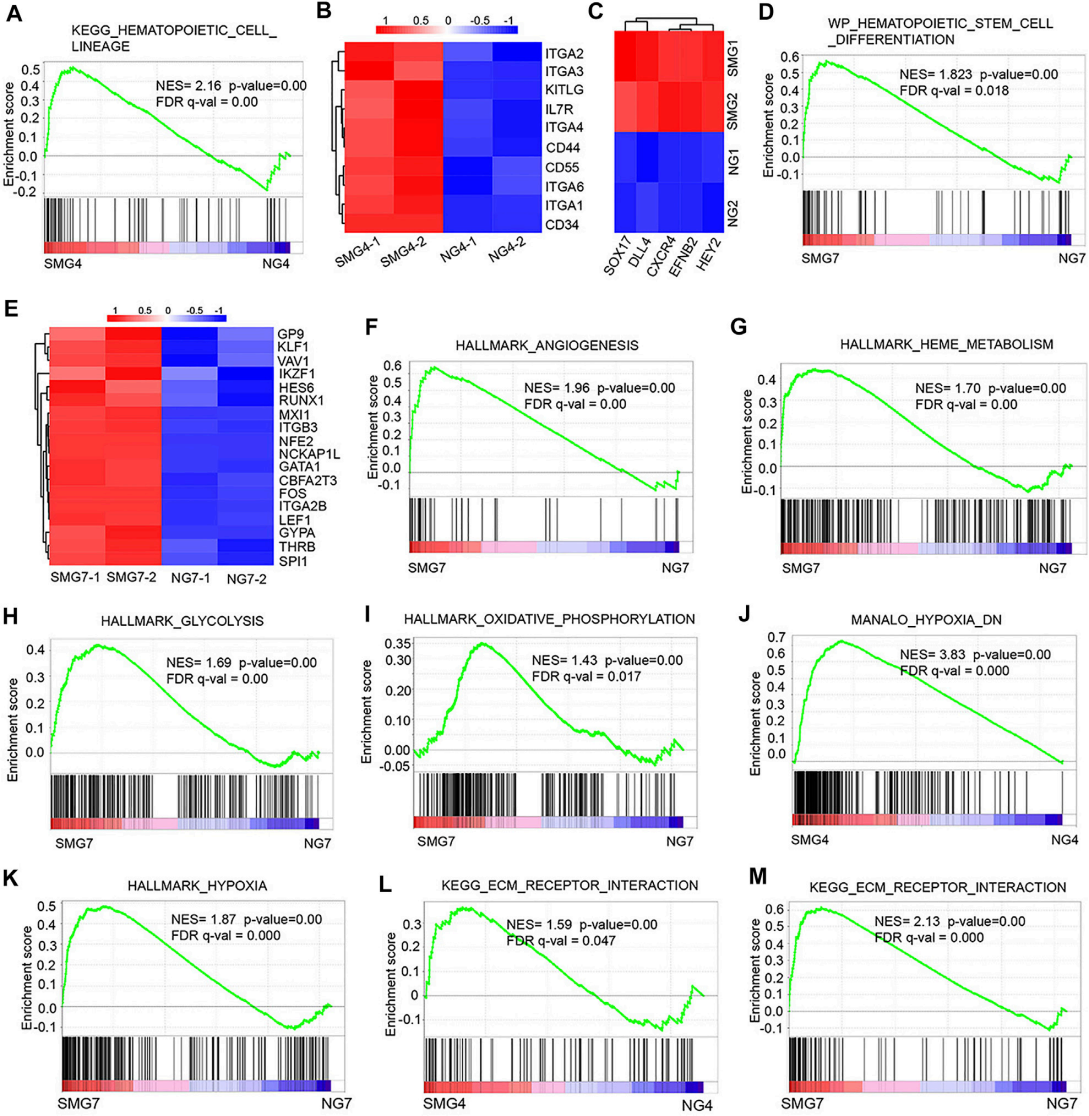
The gene set enrichment analysis (GSEA) result of DEGs in SMG vs NG cells. **(A)** GSEA of hematopoietic development on day 4 (SMG4 vs NG4) in hematopoietic cell lineage. **(B)** The heatmap shows the expression pattern of hematopoietic cell linage-related genes in SMG and NG cells on day 4. **(C)** The heatmap shows the expression of typical arterial-associated genes in SMG and NG cells on day 4. **(D)** GSEA of hematopoietic stem cell differentiation on day 7 (SMG vs NG). **(E)** The heatmap shows the expression pattern of hematopoietic stem cell-related genes in SMG and NG cells on day 7. GSEA profiles showing a significant enrichment of gene sets associate with angiogenesis **(F)**, HEME metabolism **(G)**, glycolysis **(H)** and oxidative phosphorylation **(I)** in SMG and NG cells on day 7 (SMG7 vs NG7). **(J,K)** GSEA of hypoxia-associated gene sets both enriched on day 4 and day 7 (SMG4 vs. NG4; SMG7 vs. NG7). **(L,M)** GSEA of ECM receptor interaction associated gene sets both enriched on day 4 and day 7 (SMG4 vs. NG4; SMG7 vs. NG7).

To further investigate the molecular mechanisms underlying microgravity related changes in hematopoietic development, we performed gene set enrichment analysis (GSEA). On day 4, the GSEA plot showed that a marked hematopoietic cell lineage associated genes in SMG group (Figure 4A). The KEGG pathway enrichment analysis of the DEGs showed that 10 up regulated DEGs (IL7R, ITGA6, ITGA1, CD34, ITGA3, KITLG, CD55, ITGA4, CD44, ITGA2) were enriched in hematopoietic cell lineage in SMG group (Figure 4B). Interestingly, the expression of typical arterial-associated genes, including DLL4, EFNB2, HEY2, SOX17 and CXCR4 (Lange et al., 2021), was significant in upregulation in SMG on day 4 compared with NG (Figure 4C). Moreover, we found that hematopoietic stem cell differentiation-related gene set were significantly enriched in SMG group at the culture of day 7 (Figure 4D). Key hematopoiesis-related genes were significantly upregulated in SMG differentiated cells when compared with in NG differentiated cells, including GYPA, GATA1, ITGB3, ITGA2B, RUNX1, LEF1 and NFE2 (Figure 4E), which have been reported to be essential roles for hematopoietic differentiation (Moriguchi et al., 2010; Pouzolles et al., 2016). Additionally, on day 7, gene terms were significantly enriched in angiogenesis (Figure 4F), HEME metabolism (Figure 4G), glycolysis (Figure 4H) and oxidative phosphorylation (Figure 4I). Notably, we also found some common gene sets both on day 4 and day 7 in SMG vs NG cells, which were those in hypoxia (Figures 4J,K) and ECM receptor interaction (Figures 4L,M).

### Effect of Simulated Microgravity on Different Developmental Stages of Hematopoietic Differentiation

**FIGURE 5:**
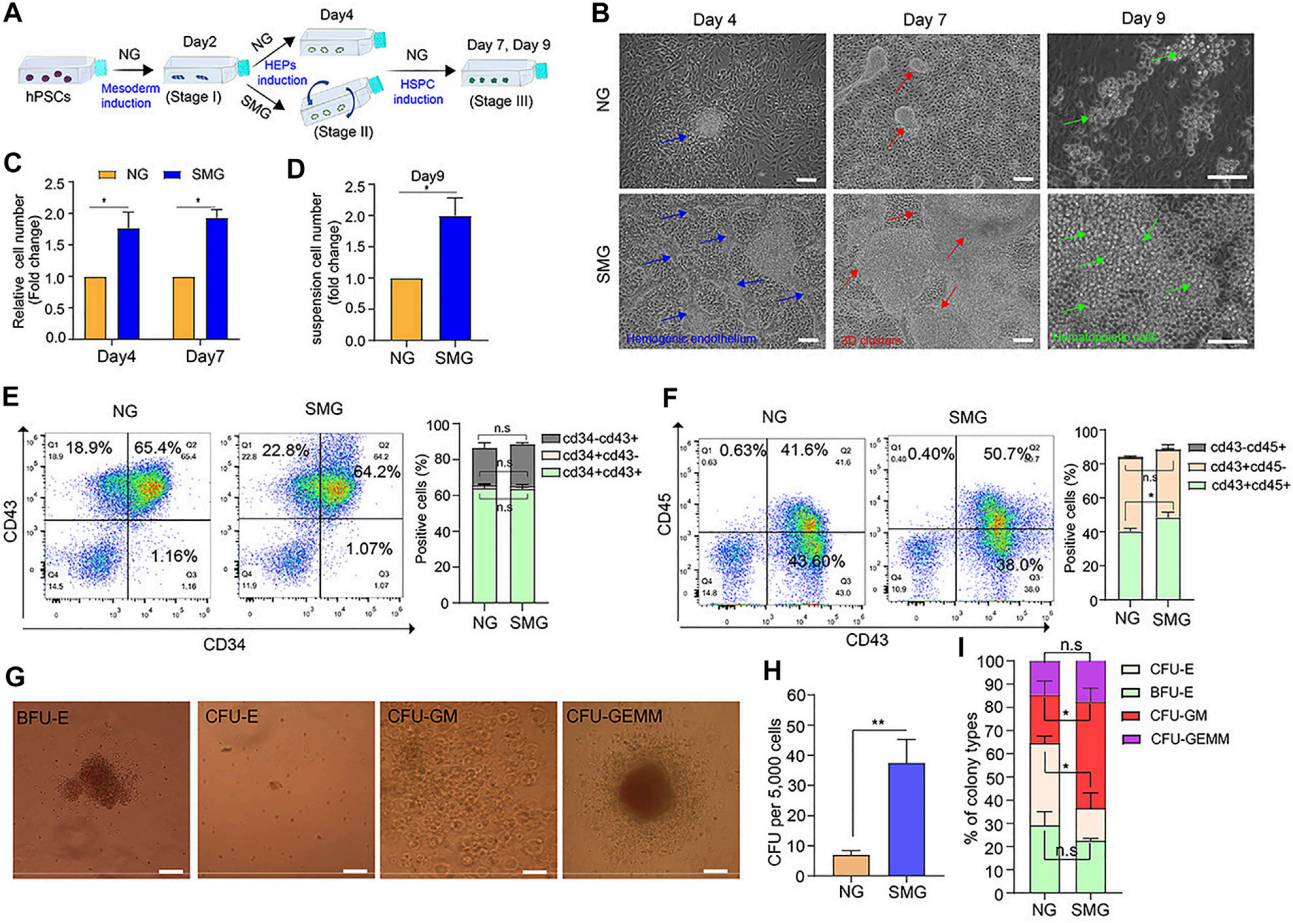
SMG promotes the hemogenic endothelium and hematopoietic development and enhances 3D cell clusters generation. **(A)** A schematic of hESC hematopoietic differentiation strategy under SMG and NG conditions. **(B)** Phase contrast image of hESC cultures on day 4 (stage 2, blue arrow indicates hemogenic epithelium like structure), 7 (stage 3, red arrow indicates hematopoietic cluster like structure) and 9 (stage 3, green cell indicates hematopoietic progenitor cells) of hematopoietic differentiation. Scale bars, 100 μm. **(C)** The relative fold change of cell number generated on day 4 and day 7. **(D)** The relative of suspension cell number generation from cell clusters on day 9 (SMG vs NG). **(E)** The percentage of percentage of CD34^+^CD43^+^, CD34^+^CD43^−^ and CD34^−^CD43^+^ cells in NG and SMG group on day 9. **(F)** The percentage of percentage of CD43^+^CD45^+^, CD43^+^CD45^−^ and CD43^−^CD45^+^ cells in NG and SMG group on day 9. **(G)** Representative colony morphologies from hESC-derived CD34^+^ progenitor cells on day 4. Scale bars, 100 μm. **(H)** The total number of colonies derived from NG group and SMG group on day 4. **(I)** The percentage of BFU-E, CFU-E, CFU-GM, and CFU-GEMM in total colonies.

To understand which stage of SMG promotes hematopoietic development, cells of the three developmental stages (mesoderm induction, HEPs induction and HSPC induction) were exposed to SMG respectively. The cell number, cell morphology and the efficiency of hematopoietic differentiation were evaluated. On day 0 to day 2 exposure with SMG (Supplementary Figure S2A), there were no significant differences in cell morphological changes (Figure S2B), the number of 3D hematopoietic like clusters (Supplementary Figure S2C) and the total cell number (Supplementary Figure S2D) in SMG compared with NG. Next, we conducted the SMG exposure at the HEPs induction stage during hematopoietic differentiation (Figure 5A). On day 4, cells differentiated in SMG exhibited more intra-aortic like structures and clusters compared with those in NG (Figure 5B). The SMG exposed cells were subsequently culture in NG conditions for hematopoietic differentiation. After 7–9 days of induction, more hematopoietic clusters and suspension hematopoietic cells appeared in SMG group than in NG group (Figures 5B–D). We then analyzed cell populations for the expression CD34, CD43 and CD45 in those suspended hematopoietic cells at day 9. We found that NG and SMG groups showed similar cell populations of CD34^+^ or CD43^+^ (Figure 5E and Supplementary Figure S3A), while SMG significantly increased the percentage of CD45^+^ cell compared to that in NG group (Figure 5F and Supplementary Figure S3B). To determine if SMG affects the number and the frequency of specific hematopoietic progenitors, we analyze the hematopoietic potential of CD34^+^ progenitors derived from hESCs on day 4 (Figure 5G). CFU assay showed SMG differentiated cells significantly increased numbers of total hematopoietic colonies with a high percentage of CFU-GM colonies compared with NG differentiated cells (Figures 5H,I). We also evaluated the role of SMG on HSPC induction from day 5 to day 8 (Supplementary Figure S4A), the results showed that cells also were prone to grow with multilayer colonies and formed many hematopoietic clusters (Supplementary Figure S4B) in SMG conditions. Additionally, the number of 3D hematopoietic like clusters was significantly increased in SMG (Supplementary Figure S4C). Taken together, these data sets demonstrated that SMG exposed at HEPs induction or HSPC induction stage could promoted the hematopoietic differentiation and facilitated the formation of 3D hematopoietic cell clusters.

### Continuously Generation of HSPC From 3D Clusters and Functionally Assessment of HSPC Derived From SMG Condition Culture

**FIGURE 6:**
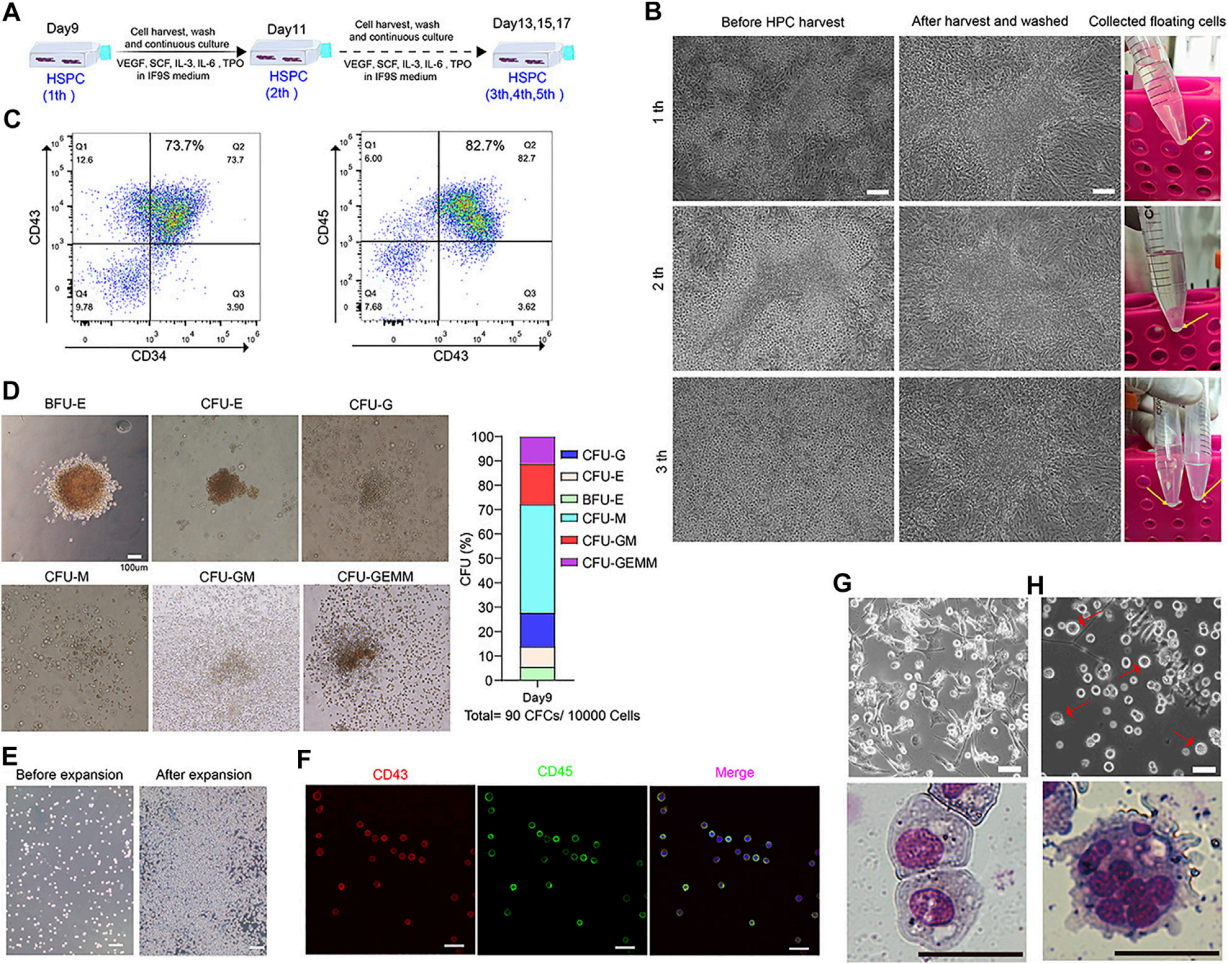
Continuously generation of HSPC from 3D clusters and functionally assessment of HSPC derived from SMG condition culture. **(A)** A strategy to continuously collection of floating cells on days 9 during hematopoietic differentiation. **(B)** Represent image of HSPCs before and after harvest from day 9 to day 13. **(C)** Flow cytometry results of CD34, CD43 and CD45 at differentiating day 11. **(D)** CFU assay of day 9 collected HSPCs and percentage of different hematopoietic colonies in MethoCult medium for 2 weeks. Scale bars, 100 μm. **(E)** Represent image of HSPCs before and after three passage expansion. Scale bars, 100 μm. **(F)** Immunostaining of CD43 and CD45 for expanded HSPCs. Scale bars, 500 μm. **(G)** Morphology of differentiated macrophages assessed by light microscopy and Giemsa staining. Scale bars, 20 μm. **(H)** Morphology of differentiated megakaryocytes by light microscopy and Giemsa staining. Scale bars, 20 μm.

By day 9, many floating HSPC accumulated around the 3D clusters regions. After harvesting, washing off the non-adherent cells and continuing to cultivate, a lot of round floating cells could be observed again from the edge of the 3D cluster region after 2 days culture (Figures 6A,B). We can harvest the floating HPSCs serval times during culturing (Figure 6B). The floating cells were harvested and checked by flow cytometry for CD34, Cd43 and CD45 at day 11, more than 80% of cells were CD34^+^CD43^+^ or CD43^+^CD45^+^ (Figure 6C). The colony-forming unit (CFU) assay showed that cells had the ability to form erythroid, granulocyte and monocyte/macrophage colonies (Figure 6D). Additionally, the collected floating cells had a robust ability of proliferation *in vitro* (Figure 6E), which showed almost of the cells was co-expressed CD43 and CD45 (Figure 6F). The expanded hESC-HSPCs were cryopreserved for further functional assessment or differentiation into functional blood cells. We also evaluated the feasibility of inducing HSPCs to differentiate into macrophages and megakaryocytes. Typical macrophage (Figure 6G) and megakaryocytes (Figure 6H) morphology could be observed after 8–10 days of differentiation with Giemsa staining.

## Discussion

Several protocols have been developed to induce hematopoietic differentiation from hPSCs *in vitro* or *ex vivo*, including co-culture with stromal cell line (for example, OP9 and AGM-S3), the formation of embryoid bodies (EBs) and in chemically defined conditions under normal gravity. However, whether microgravity is able to direct the hematopoietic differentiation of hPSCs and how is unknown. In the present study, we demonstrated that SMG facilitates hESCs differentiate to HSPC with more efficient induction of CD34^+^CD31^+^ hemogenic endothelium progenitors (HEPs) on day 4 and CD34^+^CD43^+^ HSPC on day 7, and these cells shows an increased generation of functional hematopoietic cells in CFU assay when compared with normal gravity (NG) conditions. We also found that SMG significantly increased the total number of cells on day 4 and day 7 by facilitating the formation of 3D cell clusters at stage 2 or stage 3 of HSPC induction. We performed transcriptome analysis of cells to study microgravity-associated gene expression changes and identified some key gene terms or pathways in hematopoietic differentiation under microgravity. Thousands of DEGs were identified between SMG and NG conditions. DEGs down-regulated were enriched in the axonogenesis, positive regulation of cell adhesion, cell adhesion molecule and axon guidance., while SMG resulted in the up-regulation of genes were functionally associated with DNA replication, cell cycle, PI3K-Akt signaling pathway and tumorigenesis. Additionally, some key gene terms enriched in SMG, including hypoxia and ECM receptor interaction. Importantly, HSPC obtained from SMG culture system have a robust specific ability of proliferation, formed erythroid, granulocyte and monocyte/macrophage colonies in CFU assay, and can be induced to generate macrophages and megakaryocytes. Our results showed a positive role of microgravity on hematopoietic differentiation from hPSCs and revealed an underlying mechanism for the effect of SMG on human hematopoiesis development for the first time.

Previous studies have demonstrated that cells can sense local biochemical and mechanical cues, which are critical for the control of cell behavior and embryonic development. Blood flow or low fluid shear stress has been shown to promote hematopoiesis in mouse aorta-gonads-mesonephros (AGM) and from mouse embryonic stem cells (Adamo et al., 2009; North et al., 2009; Wolfe and Ahsan, 2013; Lundin et al., 2020), which can increase the expression of Runx1 and trigger nitric oxide (NO) signaling. The previous study showed that human endothelial cells from the umbilical vein and human microvascular endothelial cells cultured in simulate microgravity conditions would produce more NO than normal gravity controls with increased levels of endothelial-nitric oxide synthase (e-NOS) and promoted angiogenesis (Versari et al., 2007), which might be mediated through PI3K-Akt-eNOS signal pathway (Shi et al., 2012). Interestingly, we also found microgravity increased the expression of Runx1 and NOS2 during hematopoietic differentiation, which might partially explain the role of SMG in promoting HSPC generation (Ran et al., 2013; Nogueira-Pedro et al., 2014). Further functional colony-forming unit experiment demonstrated that SMG increased total numbers of hematopoietic colonies in CFU assay, confirming SMG plays a positive role in early human hematopoiesis.

To identify potential key genes and pathway that are associated with enhancing hematopoietic differentiation under SMG conditions, we performed RNA-seq analysis. On day 7, GO term enrichment analysis showed that 130, 63, 71 and 41 upregulated DEGs were significantly enriched in cell adhesion, extracellular matrix organization, cell-cell signaling and angiogenesis. Similar observations were made by Wang et al. that microgravity activates Cdc42 *via* Rap1GDS1 to promote vascular branch morphogenesis (Wang et al., 2017). Through pathway enrichment, we also observed that ECM-receptor interaction, PI3K-Akt signaling pathway, pathways in cancer and focal adhesion were the most enriched pathways. We found many DEGs are integrin subunit genes, including ITGA3, ITGA4, ITGB4 and ITGB6. As previously reported that integrin-mediated signaling controls hematopoiesis *via* focal adhesion kinase (FAK) signaling or ECM assembly (Khadilkar et al., 2020). Interestingly, our GSEA analysis results shows some important metabolism pathways were enriched in SMG group, such as glycolysis and oxidative phosphorylation, suggesting that simulated microgravity significantly altered metabolism during hematopoietic differentiation. In addition, we also found some common gene sets, such as associate hypoxia and ECM receptor interaction both on day 4 and day 7 in SMG vs NG cells that might indicate the cell respond to microgravity with an increase in glucose metabolism and oxygen consumption (Uda et al., 2021).

A significant phenotype of our experiment is the discovery that SMG facilitated the formation of tridimensional clusters and favored cell proliferation by stimulated cell-matrix and cell-cell interactions (Lei et al., 2011; Costantini et al., 2019; Choi et al., 2021). Some previous studies have made efforts to promote the hematopoietic differentiation by facilitating mesoderm induction via signaling pathway modulation, including WNT activation (Wang H. et al., 2020), TGF-*β* signaling (Wang et al., 2020b) and insulin-mTOR signaling inhibition (Duan et al., 2018). Interestingly, we found that SMG enhanced hESC hematopoiesis by facilitating HEPs induction and HSPC induction, but not through increased mesoderm induction. In fact, in this study, we added GSK3 inhibitor CHIR99021 (functions as WNT activator) and TGF*β* inhibitor SB431542 in our culture system for inducing mesoderm highly efficiency with no significant difference between SMG and NG group. Continuous harvesting of HSPCs also have advantages when a large number of cells are needed, such as for cell transplantation. In our study, we can harvest the floating HPSCs serval times and allows cells to largely expanded *in vitro*. We also found that the HSPCs induced in SMG had colony-formation ability with formed typical erythroid (CFU-E), macrophage (CFU-M), granulocyte (CFU-G), granulocyte–macrophage (CFU-GM) and mixed myeloid-erythroid (CFU-mixed) colonies. Additionally, hESCs-derived from SMG generated HSPCs can be further induced to form macrophages at high efficiency. However, future functional experiments should explore whether the putative HSPC derived from hESCs under SMG conditions can reconstructing hematopoietic stem cell transplantation in immunodeficient mice.

In summary, our work reveals a previously unknown role for simulated microgravity in hematopoietic differentiation of human pluripotent stem cells. However, whether real microgravity environments have a similar impact on hematopoietic differentiation of hESCs need to be further investigated. In our next study, we will investigate the effect of microgravity on the proliferation and hematopoietic differentiation of the human embryonic stem cell aboard on China’s space station in the next 2 years, we will also combine and analyze the results from spaceflight and simulated microgravity conditions.

## Data Availability

The datasets presented in this study can be found in online repositories. The names of the repository/repositories and accession number(s) can be found below: https://bigd.big.ac.cn/gsa-human/browse/HRA001496.
