# Supplementary material for: Simulated Microgravity Potentiates Hematopoietic Differentiation of Human Pluripotent Stem Cells and Supports Formation of 3D Hematopoietic Cluster
Source: Front Cell Dev Biol. 2022 Jan 10;9:797060. doi: 10.3389/fcell.2021.797060 (PMC8784808; doi:10.3389/fcell.2021.797060)
Supplement: Supplementary file 3 [file Presentation1.PDF]

Supplemental figures

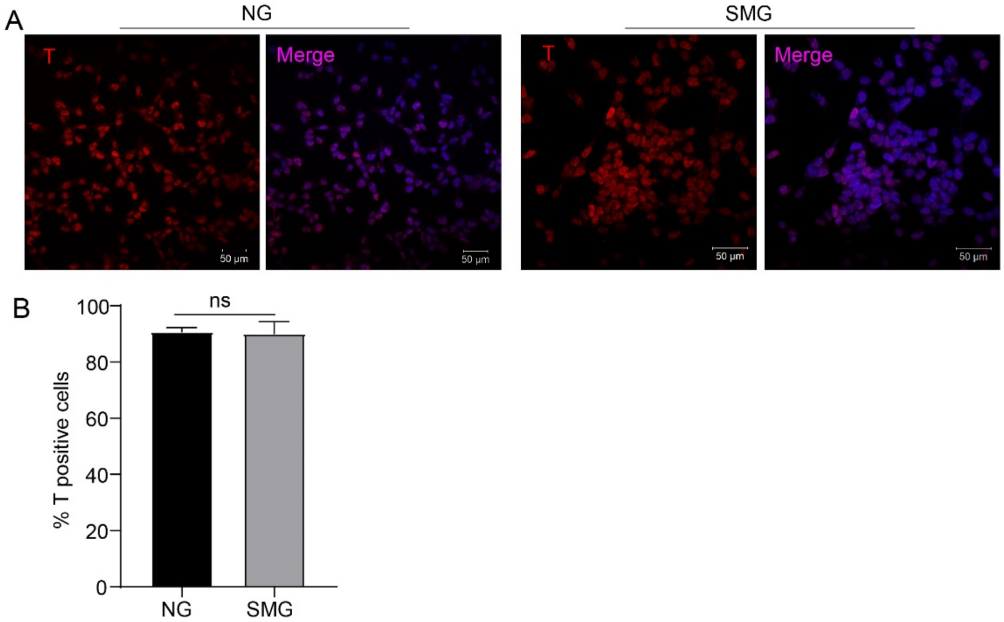

FigureS1. Differentiation of mesoderm of human embryonic stem cells (hESCs) under SMG and NG conditions. (A) Cell immunofluorescence staining for mesoderm marker T (red) at culture of day 2 under NG and SMG conditions. Scale bars, 50 µm. (B) The percentage of T positive cells in NG group and SMG group.

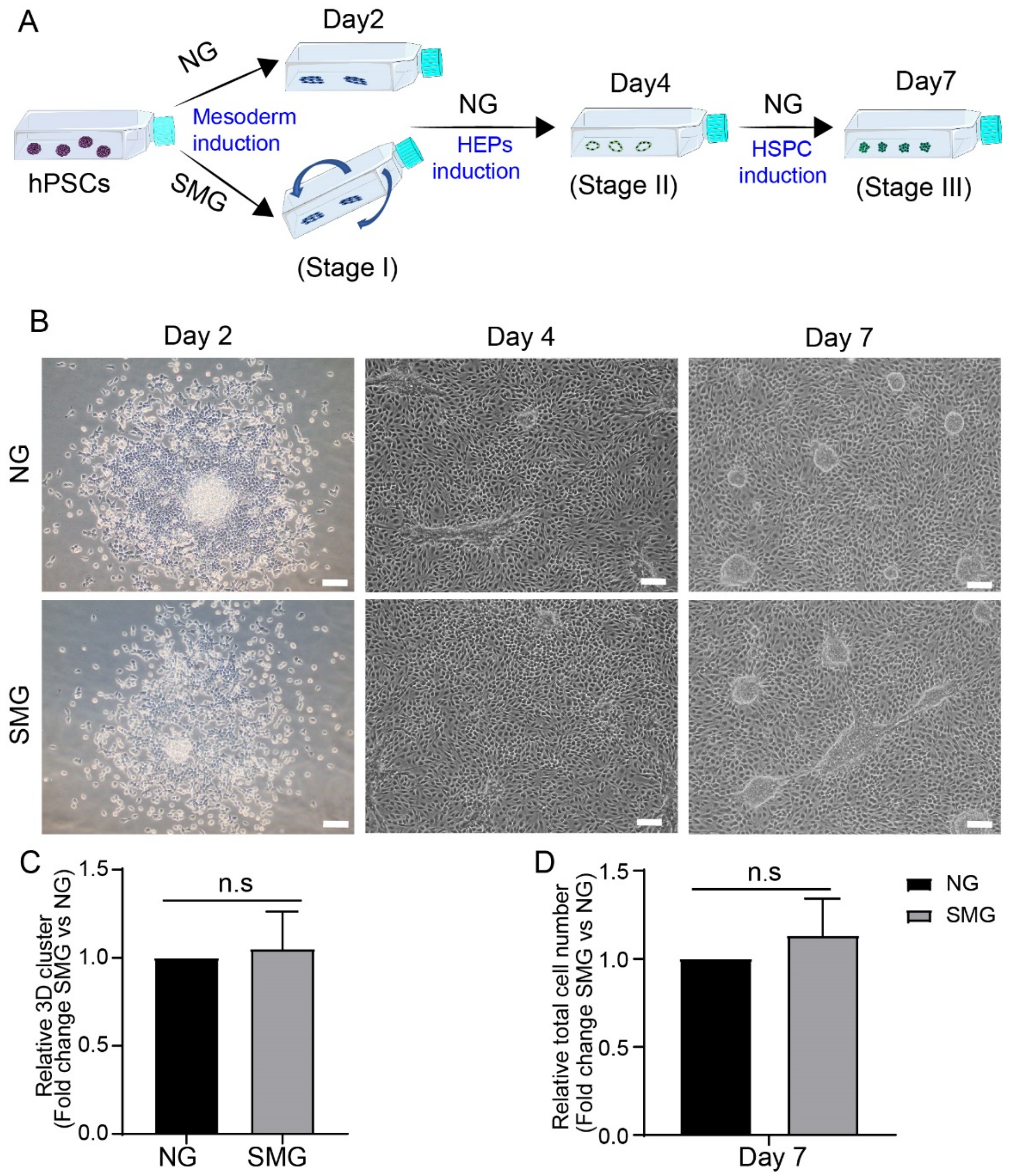

Figure S2. The effect of SMG exposure at mesoderm induction on the hematopoietic differentiation. (A) A schematic of hESC hematopoietic differentiation strategy under SMG and NG conditions. (B) Represent phase contrast image of hESC cultures on day 2, 4 and 7 during hematopoietic differentiation. Scale bars,100 μm. (C) The relative of 3D cluster generation on day 7 (SMG vs NG). (D) The relative fold change of cell number generated on day 7 (SMG vs NG).

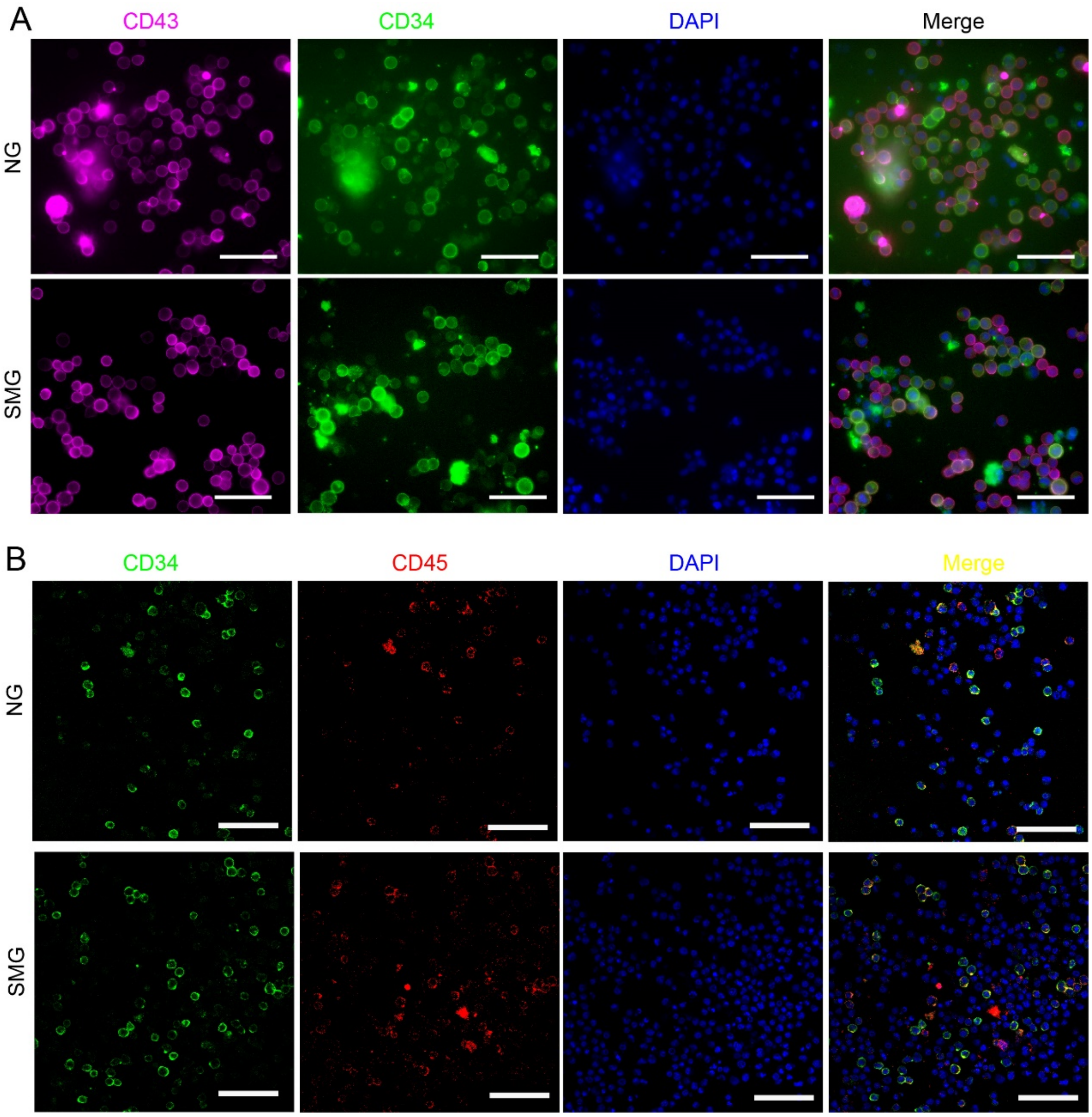

Figure S3. The effect of SMG exposure at hemogenic endothelium induction on the hematopoietic differentiation. (A) Representative immunostaining images of day 9 cells for CD34 and CD43. (B) Representative immunostaining images of day 9 cells for CD34 and CD45. Scale bars, 50 μm.

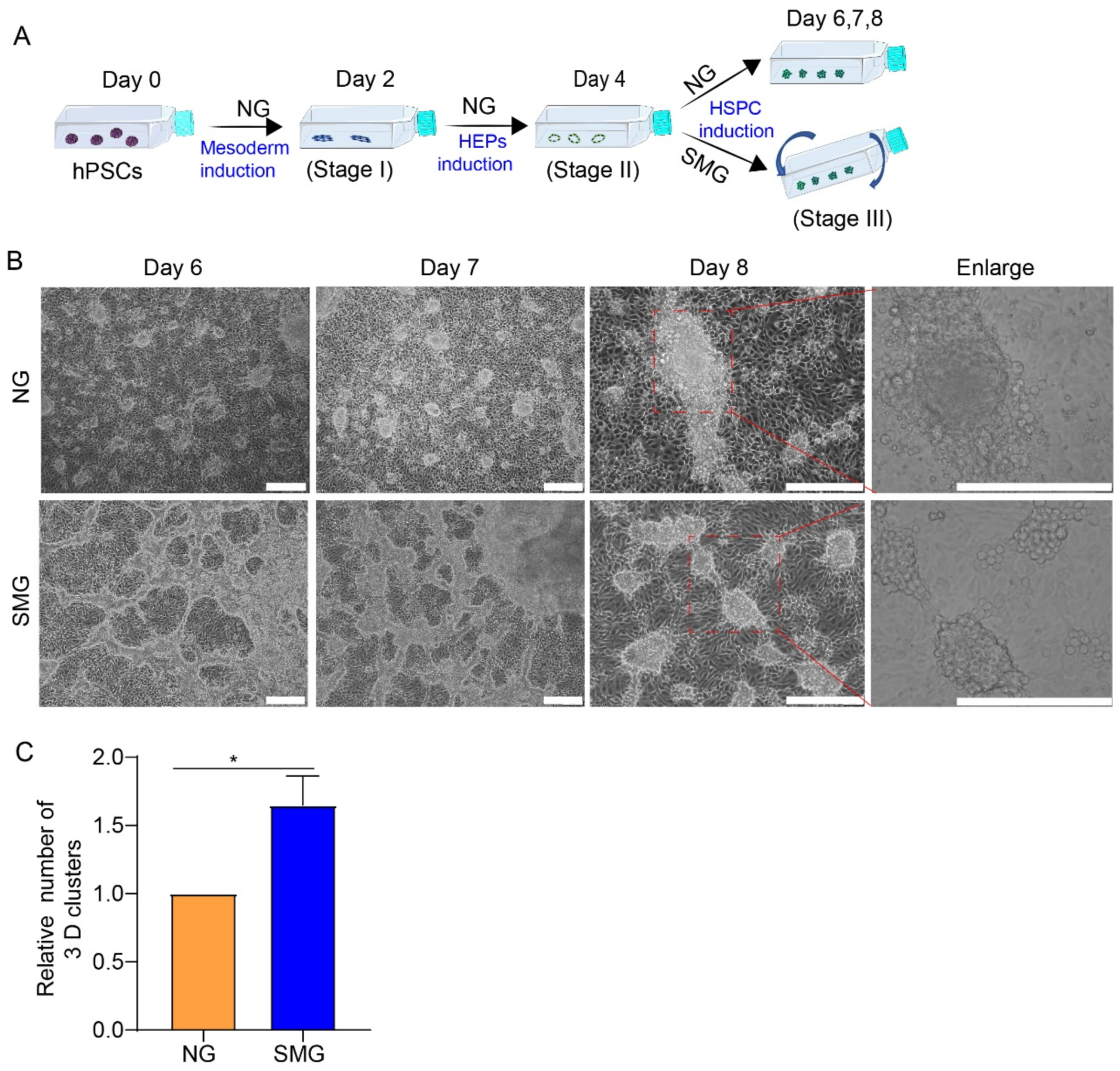

Figure S4. The effect of SMG exposure at HSPC induction on the hematopoietic differentiation. (A) A schematic of hESC hematopoietic differentiation strategy under SMG and NG conditions. (B) Represent phase contrast image of hESC cultures on day 6, 7 and 8 during hematopoietic differentiation. Scale bars,100 µm. (C) The relative of 3D cluster generation on day 7 (SMG vs NG).

Table S1. Primers for Real-time PCR

Gene	Sequence (5’-3’)
GAPDH	TGTTGCCATCAATGACCCCTT<br>CTCCACGACGTACTCAGCG
RUNX1	CTGCCCATCGCTTTCAAGGT<br>GCCGAGTAGTTTTCATCATTGCC
SOX17	GCCAAGGGCGAGTCCCGTA<br>GCATCTTGCTCAACTCGGCGTTGTGCA
NOS2	GCTCTACACCTCCAATGTGACC<br>CTGCCGAGATTTGAGCCTCATG
